# Hippocampal subfield volume differences between female-to-male transgender individuals with cross-sex hormone therapy and cisgender women

**DOI:** 10.3389/fnins.2025.1477725

**Published:** 2025-02-27

**Authors:** Gwang-Won Kim, Mina Lee, Hyun-Suk Lee, Kwangsung Park, Gwang-Woo Jeong

**Affiliations:** ^1^Advanced Institute of Aging Science, Chonnam National University, Gwangju, Republic of Korea; ^2^Department of Urology, Chonnam National University Hospital, Chonnam National University Medical School, Gwangju, Republic of Korea; ^3^Department of Radiology, Chonnam National University Hospital, Chonnam National University Medical School, Gwangju, Republic of Korea

**Keywords:** brain volume, female-to-male transgender individuals, hippocampal subfields, hormone therapy, testosterone

## Abstract

**Introduction:**

The prevalence of female-to-male (FtM) transgender individuals has risen recently, yet the effects of cross-sex hormone therapy on volumetric differences in the hippocampal subfields remain poorly understood. This study aimed to evaluate the differences of gray matter (GM) volume, especially focusing on the hippocampal subfields, in FtM transgender individuals and premenopausal cisgender women.

**Methods:**

Seventeen FtM transgender individuals who had undergone hysterectomies and were receiving testosterone therapy before participating in this study, along with 20 premenopausal women, underwent a single session of T1-weighted magnetic resonance imaging (MRI).

**Results:**

FtM transgender group had significantly higher free-testosterone (free-T) levels and lower estradiol levels compared with premenopausal women group (*p* < 0.001). In voxel-wise analysis, FtM transgender individuals showed significantly larger GM volumes in the caudate nucleus, hypothalamus, and thalamus compared with premenopausal women (*p* < 0.01, FWE-corrected). More specifically, the right hippocampal subiculum volume was larger in FtM transgender individuals (*p* < 0.05, Bonferroni-corrected), and these volumes were positively correlated with the free-T levels (*r* = 0.34, *p* = 0.04). This study revealed the specific hippocampal subfield volume differences in the testosterone-treated FtM transgender group when compared to cisgender premenopausal women group.

**Discussion:**

These findings might help elucidate the morphological variation of the specific cerebral regions associated with testosterone therapy in FtM transgender individuals and contribute to our understanding of the effects of gender-affirming hormone treatments as well.

## Introduction

Gender identity refers to one’s internal understanding and personal experience of their own gender, which may not correspond to the sex assigned at birth (Kurth et al., 2022; Klinger et al., 2023; Turnamian and Liu, 2023). Female-to-male (FtM) transgender individuals are assigned as female at birth, but identified as male (Kim et al., 2016a; Klinger et al., 2023). They often undergo medical and social transitions to align their physical appearance and gender expression with their gender identity. Recent studies (Flores et al., 2016; Meerwijk and Sevelius, 2017) have shown that the prevalence of transgender individuals in the United States is estimated between 390 and 560 adults per 100,000 in 2016.

Hormone therapy plays a central role in supporting the physical and psychological well-being of transgender individuals (Zubiaurre-Elorza et al., 2021; Turnamian and Liu, 2023). Typically, gender-affirming hormone therapy involves a combination of pharmacological interventions tailored to individual needs, which may include testosterone, progesterone, and other medications (Schneider et al., 2019; Konadu et al., 2023). Among these treatments, testosterone therapy is crucial for FtM transgender individuals to induce virilization, male-pattern body hair growth, and physical contours, as well as the cessation of menstruation (Kim et al., 2016a; Kim et al., 2016b). Testosterone therapy aids in the development of sexual characteristics and significant changes in brain function and morphometry, including alterations in brain volume and cortical thickness, suggesting a notable neuroplastic response to gender-affirming treatment (Pol et al., 2006; Zubiaurre-Elorza et al., 2014; Celec et al., 2015; Kim et al., 2015).

Previous neuroimaging studies revealed brain volume alterations in FtM transgender individuals, both in those who had not undergone hormone therapy (Simon et al., 2013; Zubiaurre-Elorza et al., 2013; Manzouri et al., 2017) and in those who had received hormone treatment (Pol et al., 2006; Zubiaurre-Elorza et al., 2014; Kim et al., 2015; Kim et al., 2019). A pioneering study (Zubiaurre-Elorza et al., 2013) demonstrated larger volume in the right putamen, which was the first to evaluate regional gray matter changes in an untreated FtM transgender group. Another study (Pol et al., 2006) found that testosterone therapy in FtM transgender individuals larger whole-brain volumes, especially in the hypothalamus, aligning more closely with typical male patterns. Another morphometric study (Kim et al., 2015) reported that the testosterone-treated FtM transgender group showed significantly larger volumes of the thalamus, hypothalamus, midbrain, gyrus rectus, head of caudate nucleus, precentral gyrus, and subcallosal area compared with female controls. Previous studies have focused on the overall brain structure, and only one study (Konadu et al., 2023) examined specific brain subregions, such as hypothalamic subunits. However, there is a significant lack of morphological studies on the subregions of the hippocampus, which are closely related to hormonal therapy in FtM transgender individuals.

Testosterone therapy is well-known for its impact on both the structure and function of the hippocampus which is a critical region for memory and emotional processing (Atwi et al., 2016; Panizzon et al., 2018; Kight and McCarthy, 2020; Choopani et al., 2023). This area is particularly sensitive to hormonal changes. A prior human study (Panizzon et al., 2018) revealed that testosterone levels were positively associated with hippocampal volume and memory performance. An animal study (Roof and Havens, 1992) using rats suggested that testosterone administration could improve the spatial navigation abilities and performance in maze tasks and, further led to an increase in the hippocampus in females. The hippocampal structure is composed of complex subfields, such as the cornu ammonis, dentate gyrus, and subiculum, and each plays a unique role and shows sensitivity to external stimuli (Filimonova et al., 2023; Konadu et al., 2023; Van der et al., 2023; Zhi et al., 2023; Long et al., 2024). We hypothesize that testosterone therapy in FtM transgender individuals may induce differences in specific hippocampal subfield volumes, which could differ from the patterns observed in cisgender women.

Identification of the specific hippocampal subfields that are susceptible to hormonal modulation might help in our understanding of the neurological aspects of gender identity, which could also lead to targeted therapeutic interventions to manage dysphoria and optimize psychological outcomes in transgender populations. However, the volumetric effects of testosterone therapy on the hippocampal subfields are not well understood.

Therefore, this study aimed to evaluate volume differences in the subcortical regions, including hippocampal subregions, in FtM transgender individuals undergoing testosterone therapy vs. premenopausal women, and assess the correlation between brain volume and sex hormone levels.

## Subjects and methods

### Ethics

The Institutional Review Board of Chonnam National University Hospital (IRB-CNUH) granted ethical approval for this study. All procedures and methods adhered to IRB-CNUH approved guidelines and regulations, with each participant providing written informed consent.

### Subjects

A total of 37 volunteers participated in this study, including 17 FtM transgender individuals (mean age: 41.1 ± 7.4 years) and 20 premenopausal cisgender women (mean age: 41.2 ± 7.5 years). All participants underwent a single magnetic resonance imaging (MRI) scan as a part of the study procedures. All participants were right-handed and were recruited through advertisements on social media and message boards.

FtM transgender individuals were selected based on several criteria (Kim et al., 2019): (1) history of hysterectomy and oophorectomy; (2) receiving testosterone therapy; and (3) no history of neurological or psychiatric illnesses. FtM transgender individuals were treated with either intramuscular injections of testosterone enanthate at a dose of 250 mg administered every 3–4 weeks, or testosterone undecanoate at a dose of 1,000 mg administered every 12–15 weeks (Gava et al., 2018; Konadu et al., 2023). The average duration of testosterone therapy in FtM transgender individuals was 7.0 ± 2.9 years. A two-step gender identity measure was used to assess the gender identity of FtM transgender individuals and cisgender women (Bauer et al., 2017). In response to the first-step question, “What is your current gender identity?” all the FtM transgender individuals selected “Transgender male.” For the second step question, “What sex were you assigned at birth, meaning on your original birth certificate?” all the FtM transgender individuals selected “Female.” Meanwhile, when the same two-step question was given to the group of cisgender women. They each responded as follows: “Female” for both the questions. The Kinsey sexuality rating scale test (0 = exclusively heterosexual; 6 = exclusively homosexual) was included as an additional measure. The average Kinsey scale rating for the FtM transgender individuals was 5.7 ± 0.5, indicating that their sexual orientation fell within the gynephilic category, meaning that they were predominantly sexually attracted to females.

Cisgender women were recruited based on the following criteria (Kim et al., 2024b): (1) absence of a menopause diagnosis as determined by Stages of Reproductive Aging Workshop (STRAW) +10 criteria; (2) regular menstrual bleeding; (3) an ovulation day based on the rhythm method; (4) no history of psychiatric or neurological illnesses; and (5) no history of hormonal, steroid, or oral contraceptive use in the month preceding the study. We included 20 premenopausal women in the premenstrual phase 10–19 days prior to their expected period.

### Serum sex hormone measurements

Serum sex hormones, including free testosterone (free-T), estradiol (E2), follicle-stimulating hormone (FSH), and luteinizing hormone (LH), were measured approximately 1 h prior to MRI acquisition and were collected between 3:00 PM and 5:00 PM. Free-T levels were measured by radioimmunoassay using a gamma counter (Cobra 5,010 Quantum, Packard Instrument Co, Meriden, CT, USA) with a Coat-A-Count free-T kit. E2, FSH, and LH levels were measured by chemiluminescent immunoassay using the ADVIA Centaur System (Bayer Healthcare, Chicago, IL, USA) and the following test kits: ADVIA Centaur E2 chemiluminoimmunoassay kit, ADVIA Centaur FSH, and ADVIA Centaur LH. Sex hormone levels were compared between FtM transgender individuals and premenopausal women using the Mann–Whitney *U* test. A Shapiro–Wilk test was used to assess the normality of the data before applying the Mann–Whitney *U* test.

### MRI data acquisition

MRI data were collected on a 3.0 Tesla Magneton Tim Trio MR Scanner (Siemens Medical Solutions, Erlangen, Germany) with a 12-channel birdcage-type head coil. T1-weighted sagittal images were acquired using a three-dimensional magnetization-prepared rapid-acquisition gradient echo (3D-MPRAGE) pulse sequence with a repetition time/echo time of 1,700 ms/2.2 ms, field of view of 256 × 256 mm^2^, matrix size of 256 × 256, and voxel size of 1 × 1 × 1 mm^3^, resulting in 176 slices.

### MRI processing and analysis

MRI data were analyzed using SPM 8 software (Statistical Parametric Mapping, Wellcome Department of Cognitive Neurology, London, UK) via diffeomorphic anatomical registration through exponentiated Lie algebra (DARTEL) analysis (Kim et al., 2024a; Kim et al., 2024b). Prior to data processing, all T1-weighted images were aligned to the anterior-to-posterior commissure line on the transverse plane. The images were then segmented into gray matter (GM), white matter (WM), and cerebrospinal fluid (CSF) using tissue probability maps based on the International Consortium of Brain Mapping (ICBM) space template for East Asian brains (ICBM152). Customized DARTEL templates of individual GM and WM images were created. All images were normalized to the Montreal Neurological Institute 152 template and then smoothed with an 8-mm full-width-at-half-maximum isotropic Gaussian kernel. The treatment of cross-sex hormones in transgender individuals has been shown to result in changes in subcortical brain areas related to memory and emotion (Holmes, 2016). Thus, for the regions of interest (ROI) analysis, we focused on seven subcortical ROIs: the amygdala, caudate nucleus, globus pallidus, hippocampus, hypothalamus, putamen, and thalamus. The masks for these ROIs were created using WFU PickAtlas software (Maldjian et al., 2003). GM volumes between FtM transgender individuals and premenopausal women in voxel-wise analysis were compared using analysis of covariance (ANCOVA) adjusted for age and whole-brain volume (family-wise error (FWE)-corrected, *p* < 0.01). A partial correlation adjusted for age and whole brain volume was used to evaluate the relationship between sex hormone levels and GM volumes.

**Figure 1:**
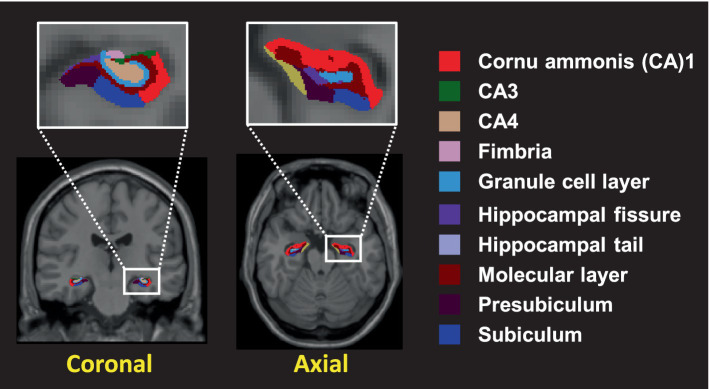
The hippocampal subfields segmented into 10 distinct different colors. Segmentation of hippocampal subfields was performed by a module built into FreeSurfer.

Hippocampal subfields were calculated using FreeSurfer v6.0 software (MGH, Boston, MA, USA). T1 image post-processing involved the following steps: the removal of non-brain tissue; the segmentation of cortical GM, subcortical WM, and deep GM volumetric structures; the triangular tessellation of the GM/WM interface and the GM/cerebrospinal fluid boundary; and topological correction (Kim et al., 2022; Kim et al., 2023). Next, automated segmentation of the hippocampal subfields was performed using a module built into FreeSurfer. Twelve hippocampal subfields were extracted for each hemisphere, including the hippocampal tail, subiculum, cornu ammonis (CA)1, hippocampal fissure, presubiculum, parasubiculum, molecular layer of the hippocampus, granular cells layer of the dentate gyrus (GC-ML-DG), CA3, CA4, fimbria, and hippocampus-amygdala transition area (HATA) (Figure 1). A multivariate analysis of variance with age as a covariate was used to compare the adjusted volumes of the hippocampal subfields of the two groups. The significance level was set to 0.05 after Bonferroni correction (the significance threshold after Bonferroni correction: *p* < 0.002). A partial correlation analysis adjusted for age and whole-brain volumes was used to evaluate the association between hippocampal subfield volumes and sex hormone levels.

## Results

### Age and serum sex hormone levels

**Figure 2:**
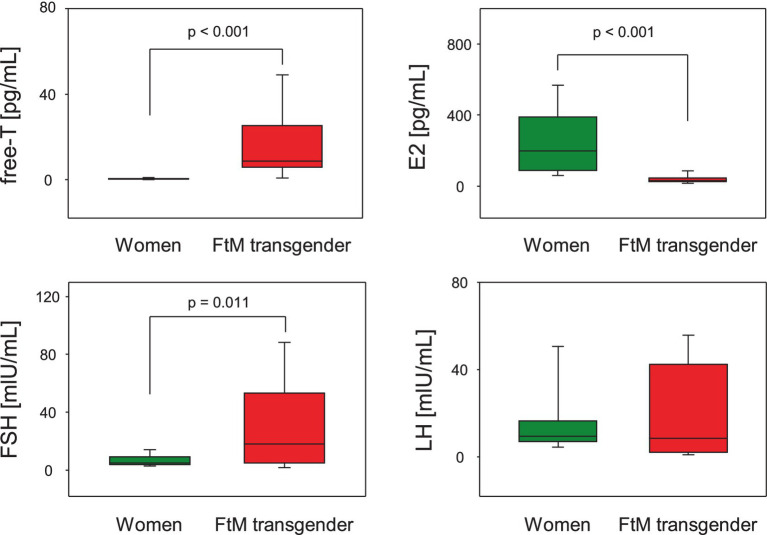
Comparison of hormone levels between premenopausal women and female to male (FtM) transgender individuals. Free testosterone (free-T), estradiol (E2), and follicle-stimulating hormone (FSH) levels were significantly different between the two groups. LH; luteinizing hormone.

No significant difference was found in age between the two groups (*p* = 0.795). The sex hormone levels in FtM transgender and premenopausal women groups are shown in Figure 2. The free-T levels in FtM transsgender and premenopausal women groups were 16.8 ± 16.9 pg./mL and 0.5 ± 0.4 pg./mL (*p* < 0.001), respectively; E2 levels were 41.3 ± 28.9 pg./mL and 259.8 ± 210.2 pg./mL (*p* < 0.001); FSH levels were 33.1 ± 32.7 mIU/mL and 6.8 ± 3.9 mIU/mL (*p* = 0.011); and LH levels were 20.3 ± 21.1 mIU/mL and 16.8 ± 16.2 mIU/mL (*p* = 0.726, Figure 2 and Table 1). Free-T and FSH levels were significantly higher, and E2 levels were significantly lower in FtM transgender group compared with premenopausal women group.

**Table 1:** Age and sex hormone levels in FtM transgender individuals and premenopausal women.

	FtM transgender individuals (*n* = 17)	Premenopausal women (*n* = 20)	*p*-value	^a^Reference ranges	
				Cisgender men	Cisgender women
Age	41.1 ± 7.4	41.2 ± 7.5	0.990	–	–
Free-testosterone (pg/mL)	16.8 ± 16.9	0.5 ± 0.4	<0.001*	Men aged 20–59: 7.2–27 pg./mL	Women aged 20–59: 0.04–2.5 pg./mL
Estradiol (pg/mL)	41.3 ± 28.9	259.8 ± 210.2	<0.001*	Men: < 52 pg./mL	Premenopausal women: 11–526 pg./mL
Follicle stimulating hormone (mlU/mL)	33.1 ± 32.7	6.8 ± 3.9	0.011*	Men aged 13–70: 1.4–18.1 mlU/mL	Premenopausal women: 1.5–33.4 mlU/mL
Luteinizing hormone (mlU/mL)	20.3 ± 21.1	16.8 ± 16.2	0.577	Men aged 20–70: 1.5–9.3 mlU/mL	Premenopausal women: 0.5–73.6 mlU/mL

Data are presented as the mean ± standard deviation. ^a^Reference ranges for sex hormones in cisgender men and women (Lee, 2007). *p*-values were calculated using the Mann–Whitney *U* test. *p < 0.05.

### Altered gray matter volumes

**Figure 3:**
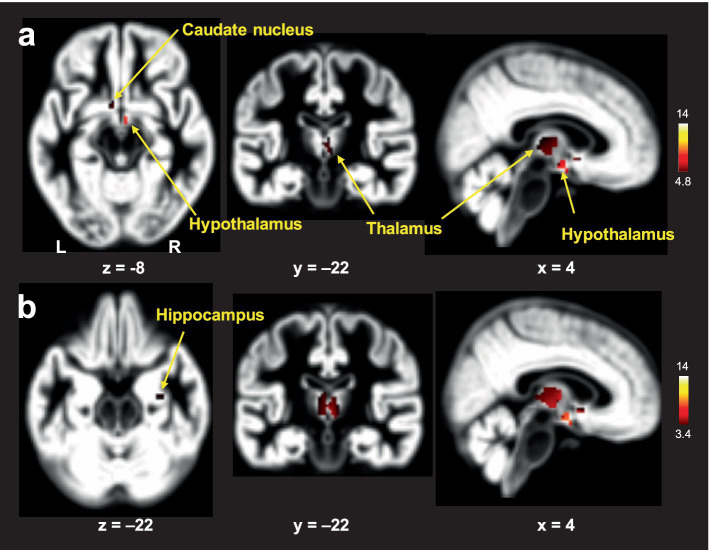
Brain areas with larger gray matter (GM) volumes in FtM transgender individuals relative to premenopausal women: analysis of covariance (ANCOVA) with age and whole-brain volume as covariates using a family-wise error (FWE)-corrected threshold of *p* < 0.01 **(A)** and an uncorrected threshold of *p* < 0.001 **(B)**. The color-coded pixels were scaled to the range (*t*-value) more than the cut-off threshold. FtM transgender individuals showed significantly lager GM volumes in the caudate nucleus, hypothalamus, and thalamus compared with premenopausal women **(A)**. In the uncorrected method, FtM transgender individuals showed significantly lager GM volumes in the caudate nucleus, hypothalamus, thalamus and hippocampus (*p* < 0.001, uncorrected) **(B)**. L; left, R: right.

**Figure 4:**
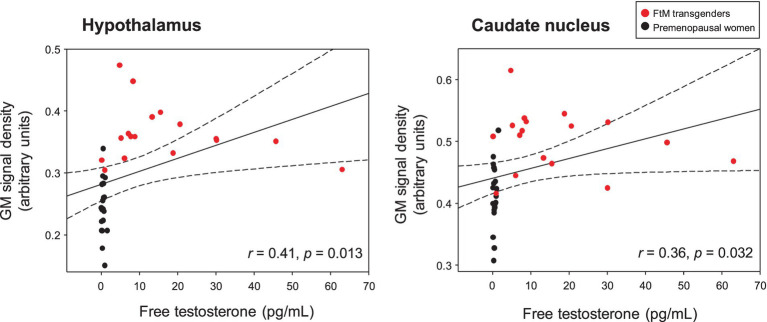
Free testosterone (free-T) levels in the two groups were positively correlated with gray matter (GM) signal density in the hypothalamus and caudate nucleus (raw values, uncorrected for age and whole-brain volume). The dotted lines show 95% confidence intervals. Free-T levels were positively correlated with GM volumes in the hypothalamus (*r* = 0.41, *p* = 0.013) and caudate nucleus (*r* = 0.36, *p* = 0.032). However, no significant correlation was found between thalamic volume and free-T levels in the two groups (r = 0.30, *p* = 0.077).

In the voxel-wise analysis, FtM transgender group showed significant larger GM volume in the hypothalamus ([x, y, z = 3, −2, −12], *t*-value = 8.7), thalamus ([x, y, z = 3, −8, 5], *t*-value = 6.7), and caudate nucleus ([x, y, z = −2, 6, −6], *t*-value = 6.4) compared with premenopausal women group (*p* < 0.01, FWE-corrected, Figure 3). No significant differences were found in GM volumes of the other ROIs between the two groups (*p* > 0.01). Free-T levels were positively correlated with GM volumes in the hypothalamus (*r* = 0.41, *p* = 0.013) and caudate nucleus (*r* = 0.36, *p* = 0.032, Figure 4). However, there was no significant correlation between thalamic volume and free-T levels in the two groups (*r* = 0.30, *p* = 0.077).

### Changes in hippocampal subfield volumes

**Figure 5:**
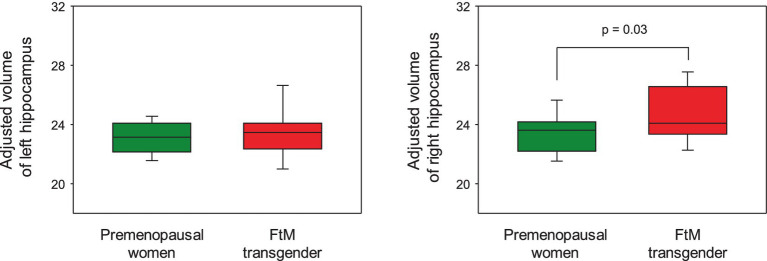
In the hippocampal regions-of-interest analysis, FtM transgender individuals showed significantly larger volume in the right hippocampus compared with premenopausal women (*p* = 0.03). However, the *p*-value was not significant at the 0.5 level (*p* = 0.002) of Bonferroni correction.

**Figure 6:**
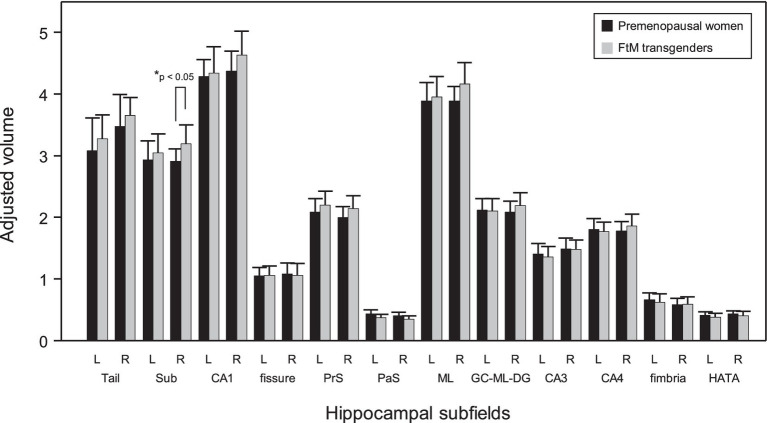
Comparison of hippocampal subfield volumes in premenopausal women and FtM transgender individuals. Sub, subiculum; CA, cornu ammonis, PrS, presubiculum; PaS, parasubiculum; ML, molecular layer of the hippocampus, GC-ML-DG; granule cell and molecular layer of the dentate gyrus; HATA, hippocampus-amygdala transition area. *significant difference (Bonferroni corrected, *p* < 0.05).

**Figure 7:**
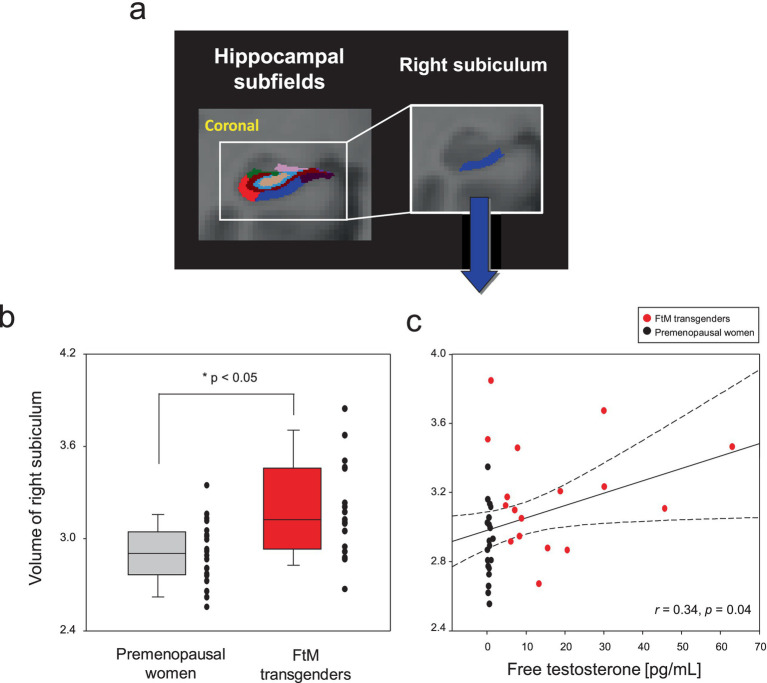
Hippocampal subfield segmentation and the right subiculum regions-of-interest **(A)**. Box and scatter plots for volumes of the right subiculum in premenopausal women vs. FtM transgender individuals **(B)**. The levels of free testosterone (free-T) in two groups were positively correlated with volumes (raw values, uncorrected for age and whole brain volume) of the right subiculum **(C)**. The right subiculum volume was larger in FtM transgender individuals compared with premenopausal women (*p* < 0.05, Bonferroni-corrected). The levels of free-T were positively correlated with volumes in the right subiculum (*r* = 0.34, *p* = 0.04). The dotted lines show 95% confidence intervals.

Although the FtM transgender group showed larger right hippocampal volumes (*p* = 0.03), the *p*-value was not significant at the 0.5 level (*p* = 0.002) of Bonferroni correction (Figure 5). Notably, the right subiculum hippocampal volume was larger in FtM transgender individuals than in premenopausal women (*p* < 0.05, Bonferroni-corrected, Figures 6, 7 and Table 2). The surface area of the other 23 hippocampal subfield ROIs was not significantly different between the two groups (all *p* > 0.05, Figure 6 and Table 2). Free-T levels were positively correlated with right subiculum volume (*r* = 0.34, *p* = 0.04, Figure 7).

**Table 2:** Comparison of hippocampal subfield volumes in FtM transgender individuals and premenopausal women.

Hippocampal subfields	Premenopausal women	FtM transgender individuals	*F*-value	*p*-value	Cohen’s *d*
L hippocampal tail	3.08 ± 0.54	3.27 ± 0.39	1.18	0.285	0.37
R hippocampal tail	3.48 ± 0.52	3.65 ± 0.29	1.25	0.272	0.38
L subiculum	2.93 ± 0.31	3.05 ± 0.30	1.63	0.211	0.43
R subiculum	2.91 ± 0.20	3.19 ± 0.31	11.19	0.002*	1.14
L CA1	4.29 ± 0.28	4.34 ± 0.43	0.30	0.585	0.19
R CA1	4.37 ± 0.32	4.63 ± 0.39	4.55	0.040	0.72
L hippocampal fissure	1.05 ± 0.14	1.06 ± 0.16	0.04	0.841	0.07
R hippocampal fissure	1.08 ± 0.18	1.06 ± 0.19	0.04	0.851	0.07
L presubiculum	2.08 ± 0.22	2.20 ± 0.23	2.21	0.146	0.50
R presubiculum	2.00 ± 0.17	2.14 ± 0.20	5.90	0.021	0.82
L parasubiculum	0.43 ± 0.06	0.37 ± 0.06	8.52	0.006	0.99
R parasubiculum	0.40 ± 0.06	0.34 ± 0.06	8.14	0.007	0.97
L molecular layer	3.89 ± 0.29	3.95 ± 0.34	0.53	0.471	0.25
R molecular layer	3.89 ± 0.23	4.16 ± 0.35	7.85	0.008	0.95
L GC-ML-DG	2.11 ± 0.19	2.10 ± 0.21	0.00	0.979	0.00
R GC-ML-DG	2.09 ± 0.17	2.19 ± 0.21	2.70	0.110	0.56
L CA3	1.41 ± 0.17	1.36 ± 0.16	0.40	0.530	0.22
R CA3	1.48 ± 0.19	1.48 ± 0.15	0.02	0.904	0.05
L CA4	1.80 ± 0.18	1.77 ± 0.16	0.18	0.671	0.14
R CA4	1.78 ± 0.16	1.86 ± 0.19	2.41	0.130	0.53
L fimbria	0.66 ± 0.12	0.63 ± 0.13	0.74	0.396	0.29
R fimbria	0.58 ± 0.11	0.59 ± 0.11	0.22	0.642	0.16
L HATA	0.41 ± 0.05	0.38 ± 0.06	2.17	0.150	0.50
R HATA	0.44 ± 0.05	0.41 ± 0.07	2.32	0.137	0.52

Multivariate analysis of variance, with age and whole-brain volume as covariates, was used to compare hippocampal subfield volumes between the two groups. Right hippocampal subiculum volumes were larger in FtM transgender individuals compared with premenopausal women (*p* < 0.05, Bonferroni-corrected). L; left, R; right, CA; cornu ammonis, GC-ML-DG; granular cell layer of the dentate gyrus, HATA; hippocampus-amygdala transition area. *Met the Bonferroni-corrected significance level.

## Discussion

In voxel-wise analysis, significantly larger GM volumes were found in the caudate nucleus, hypothalamus, and thalamus of FtM transgender individuals compared with premenopausal women, and volumes of the caudate nucleus and hypothalamus were positively correlated with free-T levels. Given that the caudate nucleus, hypothalamus, and thalamus have previously been linked to testosterone therapy in FtM transgender individuals (Pol et al., 2006; Zubiaurre-Elorza et al., 2014; Kim et al., 2015), our findings suggest that larger GM volume in these regions may be associated with the effects of testosterone.

One of the most notable findings of our study is larger GM volume in the hypothalamus of testosterone-treated FtM transgender individuals compared with premenopausal women, which is consistent with prior findings of larger hypothalamic volumes in testosterone-treated FtM transgender individuals compared with women (Pol et al., 2006; Kim et al., 2015). The hypothalamus, with high concentrations of estrogen and androgen receptors, serves as a central regulator of feedback loops controlling sex hormone homeostasis, making it particularly susceptible to the effects of sex hormones (Simerly et al., 1990; Zhao et al., 2017; Konadu et al., 2023).

The larger hypothalamic volume may reflect the hypothalamus’s sensitivity to testosterone, consistent with its known role as a key target region for sex hormones (Kim et al., 2015; Clarkson and Herbison, 2016). A recent study (Kranz et al., 2018) suggested that testosterone therapy in FtM transgender individuals alters the hypothalamic microstructure towards male proportions, supporting our observations of structural changes associated with testosterone treatment. Additionally, postmortem studies reported sex-atypical volumes and neuronal numbers in hypothalamic nuclei in the gender dysphoric brain (Zhou et al., 1995; Kruijver et al., 2000; Garcia-Falgueras and Swaab, 2008; Hoekzema et al., 2015). Thus, larger hypothalamic volumes, along with the positive correlation between hypothalamic volume and free-T levels, are likely associated with higher sex hormone levels.

The hypothalamus plays a critical role in various physiological functions, including the regulation of the endocrine system, which can be profoundly affected by sex hormones (Cornejo et al., 2016; Liu et al., 2022; Shahid et al., 2023). The presence of androgen receptors in the hypothalamus indicates that it is a target of testosterone, which can induce significant neuroplastic changes (Kranz et al., 2018; Mhaouty-Kodja, 2018). These changes may have implications for understanding the physiological underpinnings of gender identity and the effects of hormone therapy.

Our findings extend beyond the hypothalamus to the caudate nucleus and thalamus, areas linked to motor and cognitive functions, which also showed larger GM volumes in testosterone-treated FtM transgender individuals. The positive correlation between free-T levels and GM volumes in these regions suggests an association between testosterone therapy and brain structure. Further longitudinal or experimental studies are needed to determine whether testosterone therapy directly influences changes in brain structure. The caudate nucleus is involved in various functions, such as motor control, learning, and memory, and has been shown to be influenced by sex hormones (Bohbot et al., 2013; McEwen and Milner, 2017; Blanchette et al., 2020). Larger caudate nucleus volumes observed in testosterone-treated FtM transgender individuals may suggest a potential association with neuroplasticity, consistent with previous findings (Kim et al., 2015).

Similarly, the thalamus, which acts as a relay station for sensory and motor signals to the cerebral cortex, exhibited larger GM volume in testosterone-treated FtM transgender individuals (Yang et al., 2023). The involvement of this structure in cognitive processes and its sensitivity to hormonal effects suggest that testosterone treatment may enhance neural connectivity and plasticity in these regions. This observation is supported by evidence that testosterone can significantly affect brain development (Leonard et al., 2008; Rametti et al., 2012; Kim et al., 2019). The cerebral volume is 13% larger in cisgender men than in cisgender women, and the cerebellum is 10% larger (Leonard et al., 2008). Several studies (Pol et al., 2006; Zubiaurre-Elorza et al., 2014) have reported positive correlations between testosterone levels and cortical thickness in the frontal, occipital, and parietal areas. These results highlight the potential of testosterone therapy to induce broader neuroanatomical changes, including larger volumes in specific brain regions, observed in testosterone-treated FtM transgender individuals. Since other studies reported links between brain volume differences and higher free-T levels, our findings suggest that larger volumes in the caudate nucleus, thalamus, and hypothalamus may reflect morphological changes associated with higher testosterone levels in FtM transgender individuals.

Although the testosterone-treated FtM transgender individuals showed larger volume in the right hippocampus (*p* = 0.03) compared with premenopausal women, the *p*-value was not significant at the 0.05 level of FWE correction. Notably, we observed larger volumes in the right subiculum of the hippocampus in testosterone-treated FtM transgender individuals compared with premenopausal women. This finding, which was not previously reported in MRI studies of testosterone-treated or -untreated FtM transgender individuals, suggests that higher testosterone levels may exert specific effects on hippocampal subfields. The subiculum, traditionally recognized as the primary output region of the hippocampus, plays a crucial role in memory formation, spatial navigation, and stress regulation (Wozny et al., 2005; O’Mara et al., 2009). This region is also known to be sensitive to sex hormones, and a previous animal study (Pena et al., 2013) indicated that estrogen receptor protein levels were transiently elevated in the subiculum during critical developmental periods. However, the specific effects of testosterone on the subiculum have not been studied, and evidence regarding its influence on structural plasticity in this region is still limited (Hyer et al., 2018). In our study, the positive correlation between free-T levels and subiculum volumes provides compelling evidence that testosterone may play a crucial role in modulating the subiculum’s structure and function.

In cisgender men, testosterone therapy has been associated with improved cognitive function (Lasaite et al., 2017). One possible explanation for the larger right subiculum volume in testosterone-treated FtM transgender individuals is the close association with higher free-T levels, which induce plasticity. The subiculum’s involvement in cognitive functions, such as learning and memory, highlights the potential implications of these structural changes (Lipski and Grace, 2013). However, our study did not include cognitive function tests, and additional studies are needed to explore this aspect further.

The clinical implications of our findings are significant. They provide novel insights into specific hippocampal subfield volume differences in testosterone-treated FtM transgender individuals when compared to cisgender premenopausal women. These findings could be valuable for understanding the potential associations between testosterone therapy and brain structure, assisting clinicians in tailoring interventions that support both mental and physiological health. However, this study suggests that due to the lack of baseline imaging data, further longitudinal research work will be necessary to confirm our findings. Therefore, it is considered a very important study to accurately understand the morphometric variation associated with neural mechanism in FtM transgender individuals undergoing cross-sex hormone therapy.

This study had some limitations. First, the small sample size may reduce statistical power, although we addressed this by using multiple comparisons such as FWE and Bonferroni correction. Second, the absence of a male control group limited our ability to conduct comparative analyses of FtM transgender individuals and biological males, which could provide further insight into the extent of masculinization due to testosterone therapy. Future research should include hormone-treated and -untreated FtM transgender individuals to delineate the direct effects of hormone therapy. Third, our study design was cross-sectional, which limits the ability to establish causal relationships between testosterone therapy and the observed hippocampal subfield volume differences. Pre- and post-therapy imaging data must be included in longitudinal studies to clarify whether the observed differences represent therapy-induced changes or pre-existing variations. Fourth, our study did not include assessments of depressive symptoms or evaluations of the quality of life from both cohorts. Such psychological assessments would provide a more comprehensive understanding of the participants’ emotional and mental health, which could further contextualize the neurobiological findings. Fifth, the estimation of the menstrual cycle phase in premenopausal women was based on self-reported data, which may be subject to individual variations and recall bias. We did not collect additional clinical data, such as hormonal levels, to confirm or investigate the potential underlying causes of these variations in cycle length. Future studies should incorporate objective hormonal measurements to verify menstrual cycle phases and minimize potential biases.

## Conclusion

This study revealed morphometric differences in the subcortical regions, including hippocampal subfields, in FtM transgender individuals receiving testosterone therapy when compared to cisgender premenopausal women. These findings enhance our understanding of the neuroanatomical associations with testosterone levels and their role in gender-affirming hormone treatments. Further research is needed to evaluate the potential of these findings as biomarkers and their implications for optimizing therapeutic approaches to support the mental and physiological well-being of in transgender individuals.

## Data Availability

The raw data supporting the conclusions of this article will be made available by the authors, without undue reservation.
